# Curation and expansion of the Human Phenotype Ontology for systemic autoinflammatory diseases improves phenotype-driven disease-matching

**DOI:** 10.3389/fimmu.2023.1215869

**Published:** 2023-09-12

**Authors:** Willem Maassen, Geertje Legger, Ovgu Kul Cinar, Paul van Daele, Marco Gattorno, Brigitte Bader-Meunier, Carine Wouters, Tracy Briggs, Lennart Johansson, Joeri van der Velde, Morris Swertz, Ebun Omoyinmi, Esther Hoppenreijs, Alexandre Belot, Despina Eleftheriou, Roberta Caorsi, Florence Aeschlimann, Guilaine Boursier, Paul Brogan, Matthias Haimel, Marielle van Gijn

**Affiliations:** ^1^ Genomics Coordination Centre, Department of Genetics, University Medical Centre Groningen, University of Groningen, Groningen, Netherlands; ^2^ Department of Rheumatology and Clinical Immunology, University Medical Centre Groningen, University of Groningen, Groningen, Netherlands; ^3^ Department of Paediatric Rheumatology, Great Ormond Street Hospital for Children National Health Service Trust, London, United Kingdom; ^4^ Department of Internal Medicine, Erasmus Medical Centre, Rotterdam, Netherlands; ^5^ Department of Immunology, Erasmus Medical Centre, Rotterdam, Netherlands; ^6^ UOC Reumatologia e Malattie Autoinfiammatorie, IRCCS Istituto Giannini Gaslini, Genoa, Italy; ^7^ Department of Paediatric Immunology-Hematology and Rheumatology, Necker University Hospital - APHP, Paris, France; ^8^ Laboratory of Immunogenetics of Paediatric Autoimmune Diseases, UMR 1163, Imagine Institute, INSERM, Paris, France; ^9^ Department of Pediatric Rheumatology, University Hospital Leuven, Leuven, Belgium; ^10^ Division of Evolution and Genomic Sciences, School of Biological Sciences, University of Manchester, Manchester, United Kingdom; ^11^ Manchester Centre for Genomic Medicine, St Mary’s Hospital, Manchester University Hospitals National Health Service Foundation Trust, Manchester, United Kingdom; ^12^ Department of Pediatric Rheumatology, Pediatrics, Radboud University Medical Center, Nijmegen, Netherlands; ^13^ National Referee Centre for Rheumatic and AutoImmune and Systemic Diseases in Children (RAISE), Pediatric Nephrology, Rheumatology, Dermatology Unit, INSERM, Hospital of Mother and Child, Hospices Civils of Lyon, Lyon, France; ^14^ International Center of Infectiology Research (CIRI), University of Lyon, INSERM, Claude Bernard University, Lyon, France; ^15^ Division of Pediatric Rheumatology, University Children’s Hospital Basel, Basel, Switzerland; ^16^ Laboratory of Rare and Autoinflammatory Genetic Diseases and Reference Centre for Autoinflammatory Diseases and Amyloidosis (CEREMAIA), Department of Medical Genetics, Rare Diseases and Personalized Medicine, CHU Montpellier, University of Montpellier, Montpellier, France; ^17^ Inflammation and Rheumatology Section, University College London Great Ormond Street Institute of Child Health, London, United Kingdom; ^18^ Boehringer Ingelheim RCV GmbH & Co KG, Vienna, Austria; ^19^ Department of Genetics, University Medical Centre Groningen, University of Groningen, Groningen, Netherlands

**Keywords:** Human Phenotype Ontology (HPO), systemic autoinflammatory disorders (SAIDs), genome diagnostics, variant interpretation, whole exome sequencing (WES), LIRICAL

## Abstract

**Introduction:**

Accurate and standardized phenotypic descriptions are essential in diagnosing rare diseases and discovering new diseases, and the Human Phenotype Ontology (HPO) system was developed to provide a rich collection of hierarchical phenotypic descriptions. However, although the HPO terms for inborn errors of immunity have been improved and curated, it has not been investigated whether this curation improves the diagnosis of systemic autoinflammatory disease (SAID) patients. Here, we aimed to study if improved HPO annotation for SAIDs enhanced SAID identification and to demonstrate the potential of phenotype-driven genome diagnostics using curated HPO terms for SAIDs.

**Methods:**

We collected HPO terms from 98 genetically confirmed SAID patients across eight different European SAID expertise centers and used the LIRICAL (Likelihood Ratio Interpretation of Clinical Abnormalities) computational algorithm to estimate the effect of HPO curation on the prioritization of the correct SAID for each patient.

**Results:**

Our results show that the percentage of correct diagnoses increased from 66% to 86% and that the number of diagnoses with the highest ranking increased from 38 to 45. In a further pilot study, curation also improved HPO-based whole-exome sequencing (WES) analysis, diagnosing 10/12 patients before and 12/12 after curation. In addition, the average number of candidate diseases that needed to be interpreted decreased from 35 to 2.

**Discussion:**

This study demonstrates that curation of HPO terms can increase identification of the correct diagnosis, emphasizing the high potential of HPO-based genome diagnostics for SAIDs.

## Introduction

Systemic autoinflammatory diseases (SAIDs) are rare heterogeneous disorders in which there is aberrant activation of the innate immune system without an infectious cause. SAIDs are characterized by recurrent episodes of fever and other signs and symptoms of inflammation, such as arthritis, serositis, skin abnormalities, and elevated acute-phase proteins. The first monogenetic SAIDs described were hereditary fever disorders such as familial Mediterranean fever (FMF). These are caused by pathogenic variants in genes encoding for inflammasome-related proteins resulting in aberrant activation of interleukin-1 (IL1) (1, 2). Since the discovery of FMF around 1997, many more monogenic SAIDs have been described involving pathways such as the interferon-related autoinflammatory diseases (interferonopathies) and nuclear factor kappa light chain enhancer of activated B cells (NF-kB)-related autoinflammatory diseases (rhelopathies) (2, 3). The SAIDs are a heterogeneous group of rare disorders, and even though many SAIDs are monogenetic, many patients with a suspected SAID do not receive a genetic diagnosis (2, 4).

Given the rarity of these diseases, most clinicians see only a few SAID patients throughout their careers, which may lead to considerable diagnostic delay or misdiagnosis, potentially causing delayed or even incorrect treatment and adverse patient outcomes (5).

Accurate and standardized phenotypic descriptions are essential to diagnose rare diseases, discover new disease-causing genes, and make accurate phenotype–genotype correlations. This information is not only important for interpretation of genetic data but also needed for the sharing of clinical data to combine knowledge and cluster unsolved patients. Given this unmet need, the Human Phenotype Ontology (HPO) system was conceptualized and was published with initial terminology in 2008 (6). HPO is a community-based tool that has evolved as a fundamental collection of medical vocabulary, and it has been increasingly adopted as the standard to describe phenotypic abnormalities for clinical phenotypic data capture (7). Each HPO term describes a distinct phenotypic feature (e.g., lymphadenopathy, HP:0002716), and the HPO structure allows for similarity measures between patient phenotypes. HPO now contains more than 200,000 phenotypic annotations for hereditary diseases, of which 2,120 are considered rare diseases, affecting fewer than 1 in 2,000 people in the general population (5).

Unfortunately, the lack of comprehensive SAID-related HPO terms has limited the use of HPO for these diseases. In a previous endeavor, HPO terms for several known inborn errors of immunity (IEI), including 32 SAIDs, were systematically reviewed, curated, and submitted to the HPO database (8). However, whether this curation process improved the diagnosis of SAID has not been investigated. Moreover, in the period since the previous curation, 10 new monogenetic SAIDs have been discovered and submitted (9).

In this multicenter study, we aimed to demonstrate the potential of using HPO terms in diagnosing SAIDs. To start, we curated the HPO terms for the 10 new monogenetic SAIDs and submitted these to the HPO database. Next, we investigated the effect of curation of the HPO terms for all 42 curated SAIDs on diagnosis using data for 98 genetically confirmed SAID patients from eight different expertise centers. To measure the effect of the curation effort on finding the correct diagnosis, we used LIRICAL, a computational algorithm, to calculate how consistent the phenotypes are with the verified diagnosis (10). Finally, we did a pilot study to illustrate how HPO-based genome diagnostics for SAIDs could perform.

## Materials and methods

### Standardized reannotation

To curate monogenetic SAIDs, we used the 2021-10-10 version of the HPO database and a standardized reannotation method to reannotate the 10 newly discovered SAIDs (8, 11). In short, publications that described phenotypic presentations of the diseases of interest were collected by SAID experts (12). Next, phenotypic features were extracted and transformed into HPO terms using a machine learning–based model (8). Two-tier expert evaluation was performed on the HPO terms, and additional terms could be suggested as required. When at least 80% agreement was achieved, the validated terms were submitted to the HPO. Together with the results of Haimel et al. (8), 42 different SAIDs have now been reannotated. Below, we refer to the 2021-10-10 version of the HPO database as the “original set of HPO terms” and the curated version as the “curated set of HPO terms.”

### Patient cohort

For this study, we created an anonymized patient cohort of 98 patients from eight different European SAID expertise centers that are part of the ERN-RITA (European Reference Network for Rare Immunodeficiencies, Autoinflammatory and Autoimmune Diseases) (**Supplementary Table S1**). Patients who were genetically confirmed to have one of the 42 reannotated SAIDs were selected by clinicians based on availability of patients with these rare disorders in each expertise center. We aimed to include patients with as many of the reannotated disorders as possible. Clinicians were asked to retrospectively use HPO terms to describe the phenotype of the patients based on patient records. The HPO terms and the specific SAID for each patient were shared anonymously to build the resulting cohort. Below, we refer to the genetically confirmed diagnoses as “verified diseases.” If available, whole-exome sequencing (WES) data were collected in Variant Call Format (VCF) (n = 12) (**Supplementary Table S1**). The patient data fulfill all of the requirements for patient anonymity according to the ethical committee of the University Medical Centre Groningen. Written informed consent was obtained from the 12 patients for sharing their WES data.

### LIRICAL

LIRICAL (Likelihood Ratio Interpretation of Clinical Abnormalities) is an open-source program that uses the likelihood ratio (LR) statistic to determine whether a phenotypic abnormality is consistent with the diseases in the HPO database (10, 13) (**Supplementary Material 1**). We chose the LIRICAL method because it can assess combinations of HPO terms and because it outperforms similar phenotype-driven gene-prioritization methods (7). We used LIRICAL version 1.3.4.

### HPO-based LR calculation

LIRICAL calculates an LR for every candidate disease using HPO terms as input. For the calculation of the phenotype-based LR for candidate diseases per patient, LIRICAL uses the frequency of all phenotypic features among patients with a disease and the frequency in the background population. Both frequencies are extracted from the HPO database. Using the online repository and the documentation of Robinson et al. (10), we generated markdown language files (YAML) for all of the patients that contained the HPO terms documented for the patient and required data files such as the list of HPO-annotated diseases in the HPO database and the posttest probability threshold used (5%). Results were extracted from the generated Tab-Separated Value (TSV) files for all patients.

### HPO- and WES-based LR calculation

LIRICAL calculated an additional LR based on the number of predicted pathogenic variants encountered in the genes associated with the candidate disease based on HPO and the frequency of the variant in the background population. To do this, the VCF file and the documented HPO terms were used while adding the reference genome and the location of the VCF file. Using the LR, LIRICAL ranked the different possible diagnoses for every potential diagnosis reported.

### Evaluating curated HPO terms using LIRICAL

#### Comparing LRs before and after curation

LR scores for the verified diseases of the 98 patients were calculated for the verified diseases using both the original and curated set of HPO terms. The verified diseases were defined based on Online Mendelian Inheritance in Man (OMIM) disease identifiers (14). The output was matched based on the patient identifier and the OMIM identifier for their verified diseases. For comparison reasons, only patients whose verified SAIDs were found in both lists were included.

#### Comparing disease rankings before and after curation

Based on the calculated LRs, candidate diseases were ranked per patient. The assigned ranks were divided into four groups: Ranks 1–3 (high), Ranks 4–9 (intermediate), Rank ≥10 (low), and Undetected. A verified disease was considered to be undetected when it was not found in the candidate list. Using these groups, we compared the set of HPO terms before and after the curation effort. To determine whether the difference was specific for a particular SAID, we compared the number of patients for whom a specific verified SAID had a high rank [true positive (TP)] with the number of patients with a different verified SAID for which this specific SAID was also ranked high [false positive (FP)].

### Use of HPO in a genome diagnostics pilot

We also analyzed the impact of including WES data when using LIRICAL. Here, we compared the average number of candidate diseases found by LIRICAL (while still including the verified disease) with the LIRICAL results using only the HPO terms as input. As LIRICAL ranks all possible candidate diseases, whereas a clinician would initially mainly consider the highest-ranked diseases, we only included diseases found by LIRICAL with an LR >0 and a rank ≤10.

These numbers were also compared to WES analysis without HPO terms. Because LIRICAL does not support analysis of WES data without HPO terms, we used MOLGENIS VIP version 5.0.5 (default settings), a computational diagnostic pipeline that combines widely used algorithms, to annotate, filter, and classify genetic variants following the American College of Medical Genetics (ACMG) guidelines (15, 16) (**Supplementary Material 2**). To determine the number of candidate diseases, we extracted the unique number of genes in which genetic variants were found. We also carried out the same analysis using a virtual gene panel of the genes associated with the 42 different SAIDs that were reannotated in HPO. Ten patients whose verified SAIDs were found using all four methods were included.

### Statistical analysis

Assuming a normal distribution for the calculated LRs, we used the Student’s t-test to compare the average difference in LRs using the HPO terms before and after the curation effort. The Student’s t-test was used to determine the confidence interval for the average increase in LRs. To test whether this resulted in a significantly different distribution of assigned ranks, we used a two-sided Fisher’s exact test. For all tests, a *p* value <0.05 was considered statistically significant. All statistical analyses were performed in R version 4.2.2 (17).

## Results

### Reannotated diseases

For this study, we reannotated 10 newly discovered SAIDs (*SOCS1*, *TET2*, *CEBPE*, *CDC42*, *LSM11*, *RNU7-1*, *STAT2*, *RIPK1*, *NCKAP1L*, and *UBA1*) as described in the International Union of Immunological Societies (IUIS) classification criteria of 2021 (11). On average, 44 HPO terms were added to each disease. Together with 32 previously reannotated SAIDs, this resulted in 42 reannotated and curated SAIDs (**Supplementary Table S2**).

### Comparing the LRs for verified diseases before and after curation

**Figure 1:**
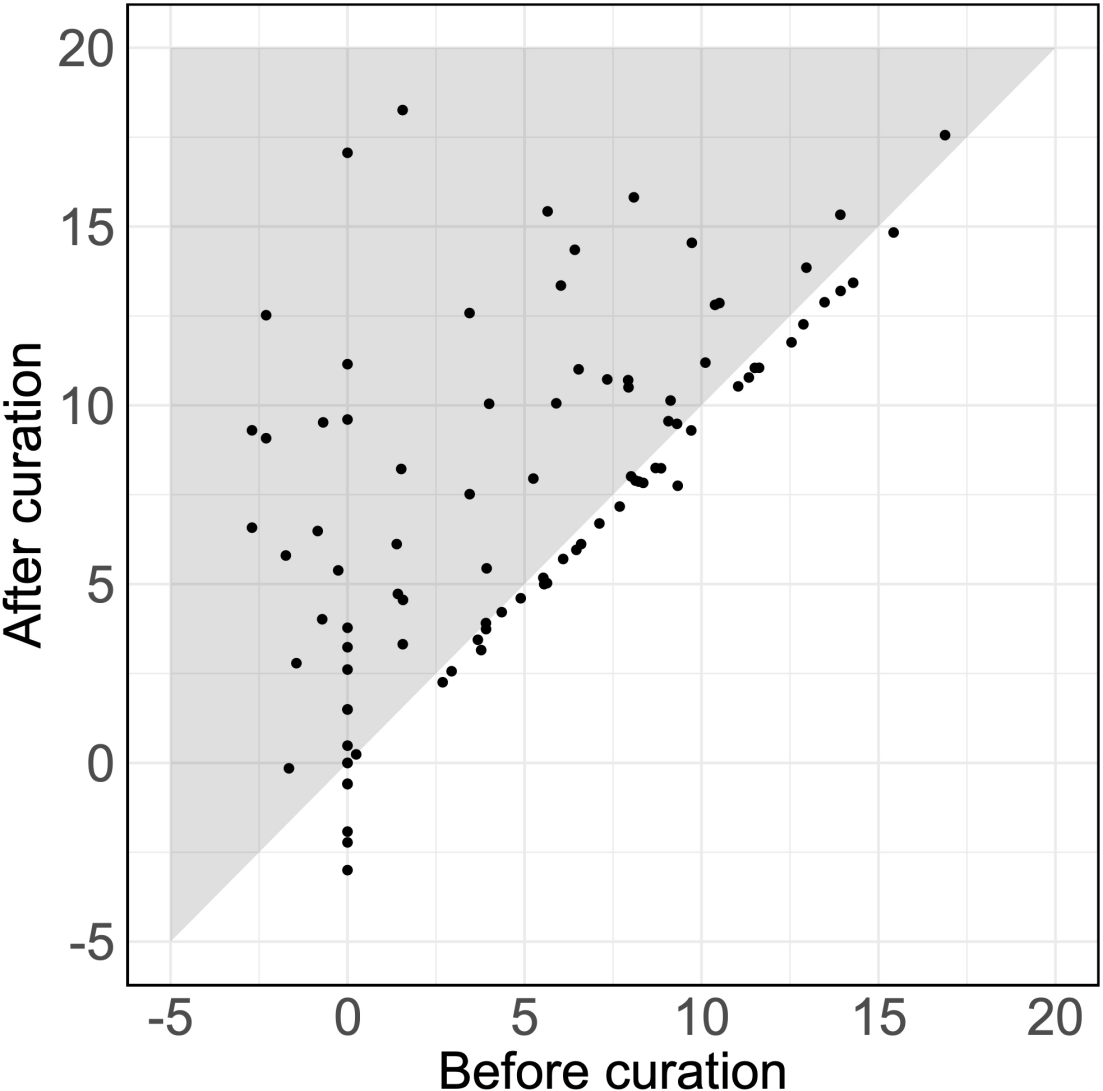
Likelihood ratios for verified diseases using HPO terms. The LRs for the SAIDs of 92 patients using the original and the curated HPO set. Each dot represents the LR of the verified SAID of a patient. The x-axis and y-axis describe the log_10_(LR)s calculated using the set of HPO terms before curation and after curation, respectively. Dots that fall within the gray zone indicate SAIDs with an improved LR. LRs can be interpreted as how many times more (or less) likely it is that patients have the disease based on the documented HPO terms as compared to patients without the disease. Negative LRs thus indicate that these patients are less likely to have the disease. Using the curated HPO set, results showed an increased log_10_(LR) (*p* = 1.7 × 10^-7^) with an average increase of 2.61 [95% CI (1.71, 3.50)].

Of 92 detected SAIDs (the SAIDs of six patients were excluded, as they were not detected using either the original or the curated set of HPO terms), 38% showed an increased log_10_(LR) with an average increase of 2.61 (*p* = 1.7 × 10^-7^) (**Figure 1**). Therefore, assuming an equal pretest probability, the curated HPO terms increased the posttest probability of the verified SAID as a diagnosis.

### Ranking of diseases before and after curation

#### Comparing the rankings of verified diseases

**Figure 2:**
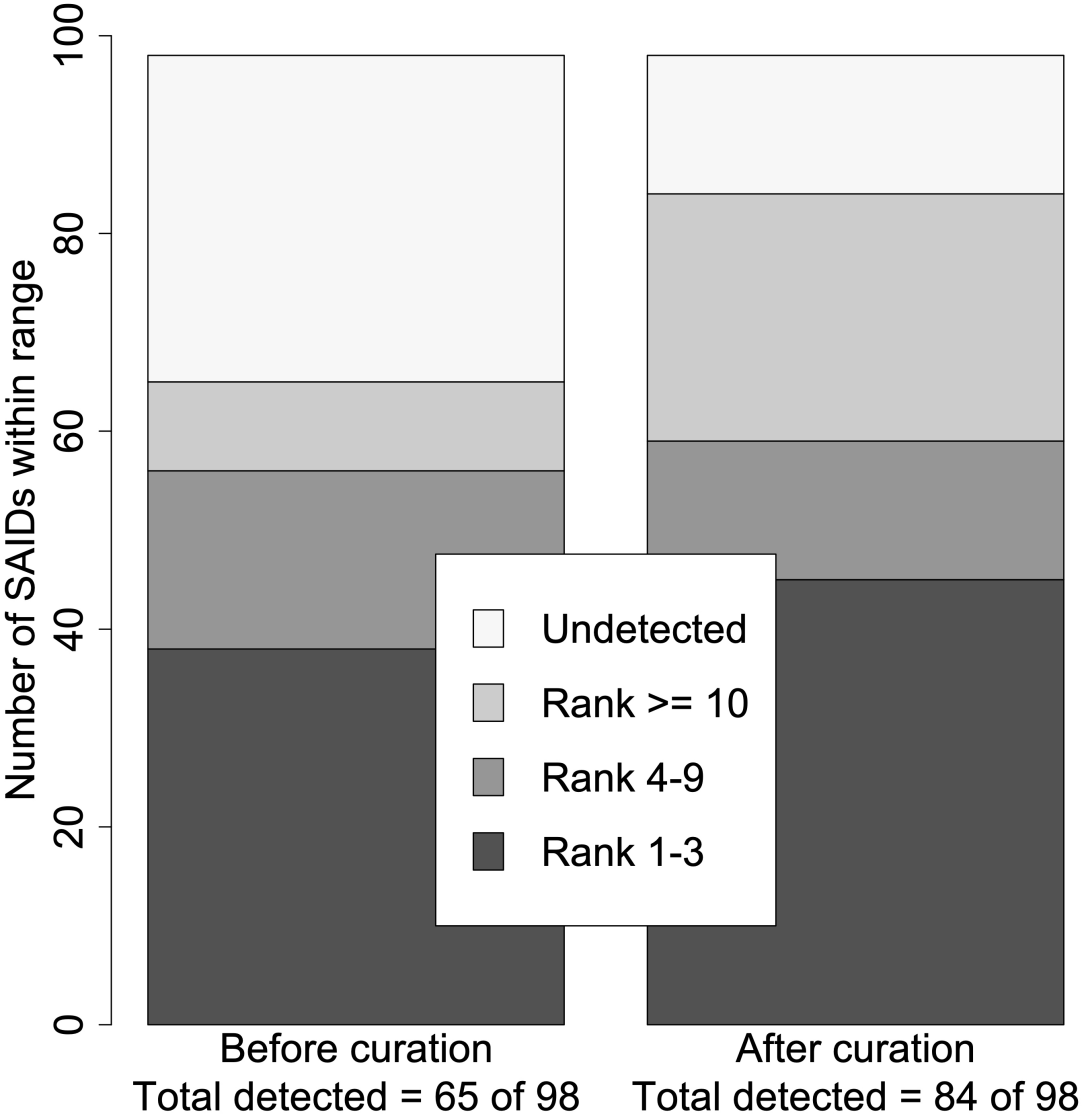
Summary of disease rank before and after curation. Using the set of HPO terms before curation, just 65 of the 98 verified SAIDs were detected (log_10_LR >0), whereas 84 of the 98 were detected using the set of HPO terms after curation. The ranked SAIDs were divided into four ranges: Ranks 1–3, Ranks 4–9, Rank ≥10, and Undetected. This resulted in two significantly different distributions within the different ranges (*p* = 0.0009). The right bar with the rank distribution using the curated SAIDs resulted in seven more SAIDs being ranked from 1 to 3. In addition, 19 more SAIDs were detected.

Next, we ranked the candidate diseases based on the calculated LRs. In general, the ranking of the verified SAID increased when using the curated set of HPO terms (*p* = 0.0009). Based on the calculated log_10_(LR)s, 66% (65/98) vs. 86% (84/98) of SAIDs were detected before and after curation, respectively. The verified SAIDs were ranked in the highest category in 38 patients using the original HPO terms, and this rose to 45 patients when using the curated HPO terms. **Figure 2** shows the total number of curated SAIDs and how they were grouped within the different ranges.

We also looked at the rankings of individual diseases and noted that the effect of the curation effort was different for each SAID. The most positive effect was seen for Muckle–Wells syndrome (MWS), where the verified SAID was assigned the highest rank in only two out of seven patients before curation as compared to six out of seven patients after curation (**Supplementary Figure S1**). In contrast, FMF was assigned a lower rank (Rank ≥10) in three out of 16 patients before curation, whereas this was the case in 12 out of 16 patients after curation.

#### Comparing TP and FP rates

To assess if the improvement after curation was specific for each SAID rather than due to improved detection of SAIDs in general, we compared the TP and FP rates. **Table 1** shows the TP and FP rates for all verified diseases assigned a rank between 1 and 3. TP rates increased for MWS, TRAPS (periodic fever, familial, autosomal dominant), and VEXAS (VEXAS syndrome, somatic), with VEXAS showing the biggest increase (from 3 out of 7 to 6 out of 7). However, the increase in specificity was different for each disease. The FP rate of MWS increased from 6 to 22, whereas the FP rate of VEXAS increased from 6 to 8.

**Table 1:** True-positive and false-positive rates.

Disease	Patients with verified SAID		Patients with other verified SAID	
	TP before curation	TP after curation	FP before curation	FP after curation
AISIMD	1 (3)	1 (3)	0	0
BLAUS	1 (3)	1 (3)	0	0
DADA2	7 (11)	6 (11)	17	9
FMF	7 (16)	3 (16)	2	2
HIDS	1 (3)	1 (3)	10	4
MWS	4 (7)	6 (7)	6	22
PAAND	0 (3)	0 (3)	1	0
PAPA	3 (3)	3 (3)	1	5
TRAPS	1 (5)	2 (5)	2	8
VEXAS	3 (7)	6 (7)	6	8
Total	28 (61) 46%	29 (61) 48%	45	58

The number of true positives (TPs) before and after curation is shown in the second and third columns, respectively, with the total number of verified cases shown in parentheses. The number of false positives (FPs) before and after curation is shown in the fourth and fifth columns, respectively. It is important to note that this is a stricter comparison of the ranking than the comparison shown in **Figure 2**, as we are only including diseases with three or more verified cases and only comparing diseases from Ranks 1 to 3 (n = 61).

### Use of HPO in a genome diagnostics pilot

For 12 of the 98 patients, we also had WES data, enabling a genome diagnostics pilot study. For the original dataset using only HPO terms, the verified SAID was found in the candidate list for nine out of 12 patients. When also using the WES data, the verified SAID was found for 10 out of 12 patients. Using the curated set of HPO terms, the verified SAID was found for 10 out of 12 patients [one patient with FMF and one patient with DADA2 (vasculitis, autoinflammation, immunodeficiency, and hematologic defects syndrome) were missed], whereas the verified SAID was found for 12 out of 12 patients when using the curated HPO terms and the WES data. Using a WES analysis detected all 12 verified SAIDs, both with and without the use of a virtual SAID gene panel of 42 different genes to filter the results (**Supplementary Table S2**).

**Figure 3:**
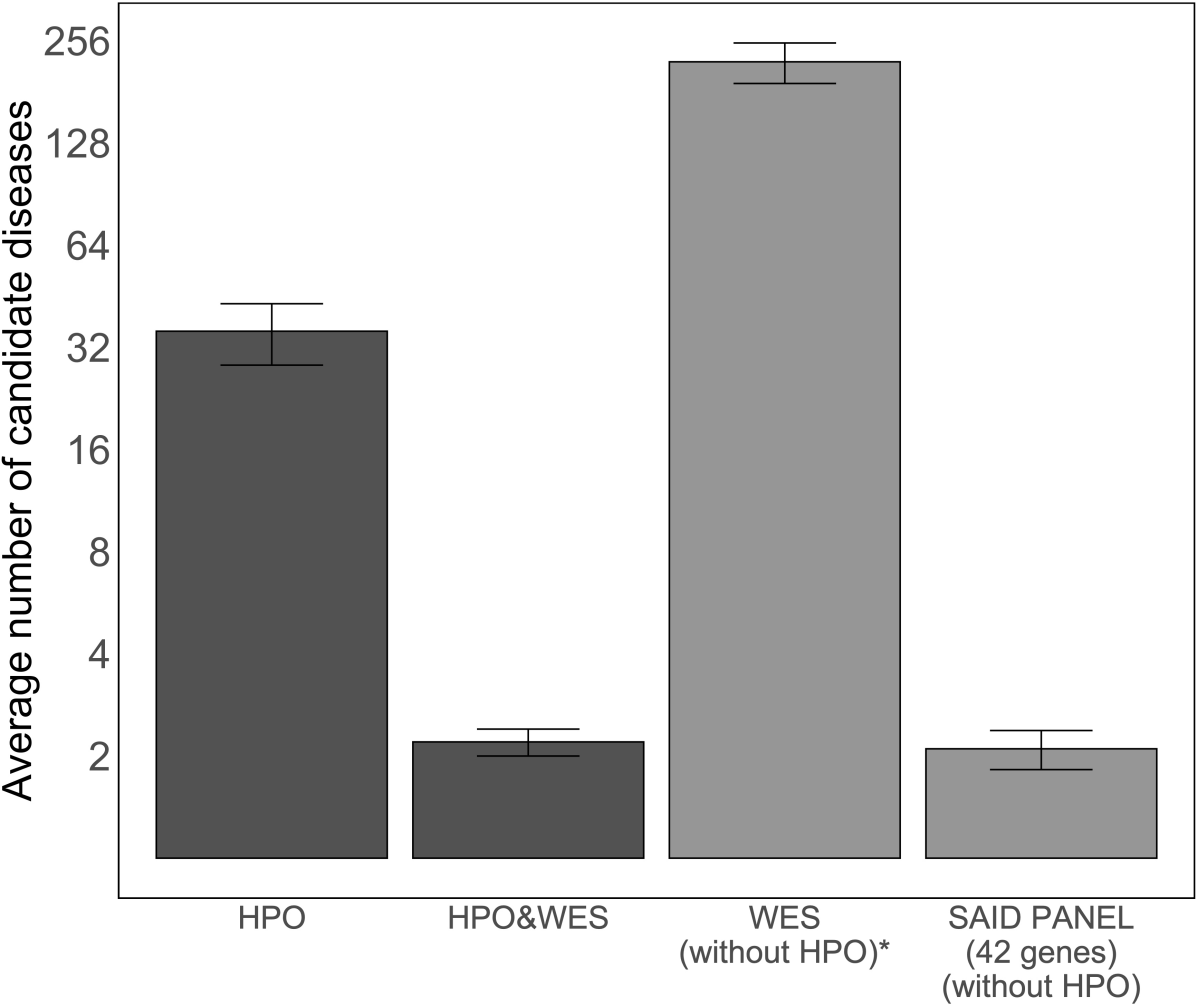
Average number of candidate diseases using LIRICAL with the curated set of HPO terms and MOLGENIS VIP. The first two bars show the average numbers of candidate diseases detected using LIRICAL with HPO without WES data and with WES data as input, 35.7 [95% CI (28.4, 43.0)] and 2.2 [95% CI (2.0, 2.4)], respectively. The total number of candidate diseases and the SAID gene panel-filtered number of candidate diseases by MOLGENIS VIP were extracted by grouping the detected variants by gene (all studied SAIDs are monogenic), and these results are represented by the last two bars, with values of 222 [95% CI (191.7, 8,252.3)] and 2.1 [95% CI (1.8, 2.4)], respectively. A log2-transformation is applied on the y-axis to compare the height of the different bars. *The number of candidate diseases is very high and is therefore not used in a diagnostic setting. This analysis setting was to demonstrate that interpreting WES data without clinical direction results in a large number of variants.

As the number of candidate diseases left to evaluate after analysis determines the applicability of an approach for genome diagnostics, we compared the average number of candidate diseases left for clinicians to interpret after the analysis using four methods: using only HPO terms, using HPO terms and WES data, using only WES data, and using WES data filtered based on a virtual SAID gene panel of 42 different genes. **Figure 3** illustrates the average number of candidate diseases that were generated. Although the numbers are small, this figure suggests that HPO-based WES-filtering produces similar results to filtering based on a SAID gene panel.

## Discussion

In this multicenter study, we aimed to demonstrate the importance of curating HPO terms for SAIDs and the potential of HPO-based SAID diagnostics. To do so, we used 42 reannotated SAIDs: 32 curated SAIDs from a previous endeavor and 10 new SAIDs that were curated and submitted to the HPO database in the current study (8, 11). To validate the curation, we collected HPO terms for 98 SAID patients from eight different European expertise centers and used LIRICAL to compare the results before and after curation (7). HPO reannotation led to enhanced homogeneity in the disease clusters and improved the ranking of the true diagnoses in most real-life patients. The curation resulted in more accurate ranking of verified SAIDs, with seven SAIDs prioritized in the highest-ranking category. In addition, we detected 19 more verified SAIDs. Finally, a pilot study showed that an analysis using curated HPO terms and WES data resulted in the highest diagnostic rate, with an average of only two candidate genes left to interpret. This indicates that curating HPO terms increases the efficiency of computational algorithms to prioritize the correct SAID in real-life patients.

After analyzing the differences in the rankings of the individual SAIDs, we observed that the effect of the curation process varied per disease. For 13 SAIDs, the total number of patients detected increased. In addition, for seven SAIDs, the number of patients assigned the highest rank increased. On the other hand, for DADA2 and FMF, the number of patients detected did not change and the average rank decreased. The same variation in the effect of the curation process was reflected by differences in the TP and FP rates for different verified diseases, which indicates how well LIRICAL is able to rank the diseases using the LR. TP rates increased for MWS, TRAPS, and VEXAS, with VEXAS showing the biggest increase, from 3 out of 7 to 6 out of 7. However, the increase in specificity is also different for each disease. The FP rate of MWS increased from 6 to 22, whereas the FP rate of VEXAS increased from 6 to 8. Additionally, although the TP for DADA2 decreased slightly, from 7 to 6, the FP rate for DADA2 decreased from 17 to 9. This suggests that an increase in sensitivity could be at the expense of a decrease in specificity. However, in clinical use, high sensitivity for potential rare disorders might be more valuable for drawing the attention of clinicians to diseases they did not encounter before in regular practice.

One possible explanation for the variation per disease could be the homogeneity of disease phenotypes in SAID patients, which may have led to overlap between the different documented HPO terms. This phenotypic overlap could dilute the contribution of an individual HPO term to the LR of a specific verified disease. This effect is also represented by the slight drop in the diagnoses below the diagonal in **Figure 1** and by the fact that the pilot study missed DADA2 and FMF, which are both annotated with many common HPO terms. A technical explanation for this apparent lack in specificity could be that LIRICAL assumes an equal pretest probability for all SAIDs (10). However, in practice, it seems more likely that clinicians have a suspicion for a specific subset of diseases based on their experience. Finally, LIRICAL assumes that there is no relation between phenotypes and that they always occur independently. Nevertheless, phenotypic features could include or exclude each other, contributing to more specific HPO term annotations.

The diagnostic value of a computational algorithm is determined by both the prioritization of candidate diseases and the number of candidate diseases left to evaluate. The latter number represents the number of diseases left for clinicians to interpret to verify their suspicions of a possible diagnosis or to apply for a SAID gene panel study. Therefore, we compared the average number of candidate diseases in the list after analysis using different methods. The number of candidate diseases found using HPO-based WES analysis was similar to the number of candidates found using a SAID-based virtual gene panel analysis. Although we were only able to collect WES data for 12 patients, our results suggest that the added value of HPO terms is most prominent when using them in genetic analysis, as also concluded by Yuan et al. (7).

Our study has some limitations. Although we involved pediatric and adult expertise centers from different countries, SAIDs are rare diseases, so we could have introduced selection bias due to the limited availability of patients in the different centers. Another source of bias could be introduced by the individual clinicians who retrospectively translated the clinical description of the patients into HPO terms. Different clinicians might have a tendency to assign specific HPO terms or to use a greater number of HPO terms to describe a patient phenotype. For example, the minimum number of assigned HPO terms to an MWS patient is 3 and the maximum number of assigned HPO terms to an MWS patient is 12. A solution here could have been to use a predetermined method, e.g., a two-tier expert evaluation, to assign HPO terms. However, our aim was to make use of the diversity in clinicians to evaluate the added value of HPO terms in daily practice. For this reason, we did not specify the method by which the HPO terms needed to be assigned.

The HPO system itself is a community-based system in which clinicians and translational researchers can extend and refine the HPO system (6). It combines the experience and knowledge of experts all over the world. However, it is dependent on the quality of many different sources and the availability of HPO terms for specific symptoms. For some relevant SAID symptoms, the HPO terms were unavailable, such as non-infectious osteomyelitis. These new HPO terms have been submitted to the HPO project but were not yet included in the current study. In addition, because of the rarity of many SAIDs, we did not use the frequency of symptoms and age of onset in the annotation of the specific SAIDs. For less rare SAIDs like FMF, including this information might have resulted in a better prioritization and detection rate.

We chose to use LIRICAL to measure the impact of the curation because it was developed by the same research group that initiated the HPO project and because the open-source LIRICAL software is publicly available (6, 10). Additionally, LIRICAL provides the possibility to use multiple HPO terms and the combined information of HPO terms and WES data. However, we have not performed a benchmark study to compare the different available phenotype-based prioritization tools that might perform better in the prioritization of the correct disease (18). Nevertheless, we have shown that systemically curating HPO terms for SAIDs significantly improves the ranking of the correct diagnoses in real-life patients and might accelerate clustering of phenotypes in larger disease cohorts.

A potential use of HPO terms in genome diagnostics could be as a method for developing WES gene panels. Gene panel–based filtering for genetically heterogeneous disorders is widely implemented in current practice (19). Advantages of this approach are its focus on well-defined genes and its ability to minimize incidental findings. Nevertheless, because of their focus, gene panels may miss important disease-related genes that were initially not related to the disease (20). Moreover, selection and curation of adequate target genes for a panel are time-consuming. Hereditary genetic disorders can affect more than one organ system, with various clinical presentations, which makes gene panel curation a challenging task (19). As a consequence, bioinformatic approaches have been developed to create phenotype-driven gene targets. Maver et al. (19) demonstrated that phenotype-based associations between genes in a virtual gene panel correspond with the gene associations in genome diagnostics and that it could be integrated into sequencing workflows. Using a phenotype-based approach could save time when curating new gene panels, as clinical experts and researchers from different disciplines are able to contribute to the development of the HPO database (21). Finally, because genes can be related to multiple HPO terms, developing a virtual gene panel based on combined sets of HPO terms that are related to specific diseases could increase diagnostic specificity.

In conclusion, we have demonstrated the potential of HPO-based SAID diagnostics and the value of our HPO curation effort by an increased probability of finding the correct diagnosis in real-life patients. Future curation efforts could contribute to the annotation of more specific HPO terms to distinguish between homogeneous disease phenotypes for SAIDs and other disease clusters. This will support the identification of new disease-causing genes and potentially lead to high-quality phenotype-based virtual gene panels.

## Data availability statement

The original contributions presented in the study are included in the article/**Supplementary Material**. Further inquiries can be directed to the corresponding author.

## Ethics statement

The studies involving humans were approved by the ethical committee of the University Medical Centre Groningen. The studies were conducted in accordance with the local legislation and institutional requirements. Written informed consent from the 12 patients for sharing their WES data was obtained.

## Author contributions

Wrote the paper: WM, GL, OC, MH, MvG. Curated HPO terms for SAIDs: GL, OC, PD, MG, BB-M, CW, TB, EH, AB, DE, RC, FA, PB. Analyzed the data: WM, MH. Conceived and designed the experiments: WM, GL, MvG, MH, JV, LJ, MS. Supervised project: MvG. Gathered patient data: GL, PD, EO, GB, EH, MG, RC, CW, FA, BB-M. Reviewed paper: GL, OC, MG, MH, PD, MG, BB-M, CW, TB, LJ, JV, MS, EO, EH, AB, DE, RC, FA, GB, PB. All authors contributed to the article and approved the submitted version.

## Glossary

**Table:**

HPO	Human Phenotype Ontology
IEI	inborn errors of immunity
SAID	systemic autoinflammatory disease
WES	whole-exome sequencing
LIRICAL	Likelihood Ratio Interpretation of Clinical Abnormalities
NGS	next-generation sequencing
VIP	Variant Interpretation Pipeline
DTA	Data Transfer Agreement
LR	likelihood ratio
VCF	Variant Call Format
HTML	Hyper Text Markup Language
TSV	Tab-Seperated Value
YAML	Yet Another Markup Language
B	benign
LB	likely benign
VUS	variant of unknown significance
LP	likely pathogenic
P	pathogenic
IUIS	International Union of Immunological Societies
CI	confidence interval
AFND/PAAND	neutrophilic dermatosis, acute febrile
AGS1	Aicardi–Goutières syndrome 1
AGS8	Aicardi–Goutières syndrome 8
AGS9	Aicardi–Goutières syndrome 9
AIADK	vitiligo-associated multiple autoimmune disease susceptibility 1
AIEFL	autoinflammation with episodic fever and lymphadenopathy
AIFEC	autoinflammation with infantile enterocolitis
AILJK	autoimmune interstitial lung, joint, and kidney disease
AIPDS	autoinflammation, panniculitis, and dermatosis syndrome
AISBL	autoinflammatory syndrome, familial, Behcet-like
AISIMD	autoinflammatory syndrome, familial, with or without immunodeficiency
BLAUS	Blau syndrome
CINCA	CINCA syndrome
DADA2	vasculitis, autoinflammation, immunodeficiency, and hematologic defects syndrome
DIRA	interleukin 1 receptor antagonist deficiency
DITRA	psoriasis 14, pustular
FCAS1	familial cold inflammatory syndrome 1
FCAS4	familial cold autoinflammatory syndrome 4
FMF_AD	familial Mediterranean fever, AD
FMF_AR	familial Mediterranean fever, AR
HIDS	hyper-IgD syndrome
HLPS	histiocytosis-lymphadenopathy plus syndrome
IMD72	immunodeficiency 72 with autoinflammation
IMD75	immunodeficiency 75
MAJEED	Majeed syndrome
MEVA	mevalonic aciduria
MWS	Muckle–Wells syndrome
NISBD1	inflammatory skin and bowel disease, neonatal, 1
PAPA	pyogenic sterile arthritis, pyoderma gangrenosum, and acne
PDR	pigmentary disorder, reticulate, with systemic manifestations, X-linked
PLAID	familial cold autoinflammatory syndrome 3
PRAAS1	proteasome-associated autoinflammatory syndrome 1 and digenic forms
PSORS15	psoriasis 15, pustular, susceptibility to
PSORS2	psoriasis 2
PTORCH3	pseudo-TORCH syndrome 3
SAVI	STING-associated vasculopathy, infantile-onset
SGD1	specific granule deficiency
SPENCDI	spondyloenchondrodysplasia with immune dysregulation
TKS	Takenouchi–Kosaki syndrome
TRAPS	periodic fever, familial, autosomal dominant
VEXAS	VEXAS syndrome, somatic
cherubism	cherubism
