# Supplementary material for: Curation and expansion of the Human Phenotype Ontology for systemic autoinflammatory diseases improves phenotype-driven disease-matching
Source: Front Immunol. 2023 Sep 12;14:1215869. doi: 10.3389/fimmu.2023.1215869 (PMC10536149; doi:10.3389/fimmu.2023.1215869)
Supplement: Supplementary file 5 [file DataSheet_1.docx]

**Supplementary data**

1. **LIRICAL**

We used LIRICAL (LIkelihood Ratio Interpretation of Clinical AbnormaLities), a computational algorithm that utilises the likelihood ratio statistic (LR), to determine the effect of curated Human Phenome Ontology (HPO) terms on phenotype-driven genomic diagnostics (1). Within LIRICAL, the LR is the probability of a set of phenotypes being present in a patient with a specific disease divided by the probability of having the same result in a patient with a different disease. LIRICAL uses this statistic to determine whether each phenotypic abnormality is consistent with the diseases in the HPO database.

LIRICAL calculates the LR of each phenotypic feature observed for a patient. In this model, each recorded phenotypic observation is defined as a clinical test. The numerator is the frequency with which each phenotype is encountered in affected patients determined by computational disease models of the HPO project. When this information is not available, a default frequency of 100% is used. The denominator is the probability that the phenotypic feature is associated with a disease different than the disease in the numerator. This is estimated as the summation of the frequencies of phenotype associations with all other diseases, assuming an equal pre-test probability for all diseases.

If a gene associated to a disease is known, the patient’s genotype is also used as a diagnostic test in the calculation of the LR statistic. LIRICAL defines the genotype as the combination of variants in the disease-associated gene. This genotype LR aims to model the number of pathogenic variants and the probability that the allele is disease-causing. The model is created based on the mode of inheritance, the expectation of observing one or two pathogenic alleles and the probability of a variant in the background population using a compound Poisson and Bernoulli distribution. To determine the pathogenicity of variants, LIRICAL uses Exomiser, a computational algorithm that prioritises disease-causing variants using a pathogenicity score based on conservation and the physicochemical properties of the encoded protein (2).

Finally, based on the composite phenotype and genotype LR (or only on the phenotype LR when no genotype is available), LIRICAL ranks the different possible diagnoses and calculates a post-test probability for every potential diagnosis reported.

For our study we used LIRICAL version 1.3.4, which can be downloaded from <https://github.com/TheJacksonLaboratory/LIRICAL>.

1. **Variant classification by MOLGENIS VIP**

The MOLGENIS VIP pipeline uses a module in which it uses different databases and computational algorithms to annotate, prioritise and filter genetic variants in VCF files. To annotate variants and prioritise them accordingly, MOLGENIS VIP uses sources such as the ENSEMBL Variant Predictor (VEP), ClinVar, VKGL, GnomAD, AnnotSV and CAPICE (3, 4, 5, 6, 7). After MOLGENIS VIP has annotated the VCF files, it uses a decision tree to filter and prioritise the different variants. Finally, based on a predefined decision tree, all variants are classified as likely pathogenic (LP), pathogenic (P), benign (B) or likely benign (LB).

For our study, we used version MOLGENIS VIP 5.0.5 with the default decision tree for genome build GRCh37 for gene variant classification. First, variants that have been annotated with a LP or P classification in the ClinVar or VKGL database were also classified as LP or P by MOLGENIS VIP, while LB and B variants were filtered out. Subsequently, variants with a GnomAD mean allele frequency (MAF) ≥0.02 that have been previously classified as LB or B by ClinVar and VKGL are filtered out. Next, variants with an AnnotSV classification LP or P are classified accordingly, and variants with an AnnotSV classification of B are filtered out. Variants that are classified as a variant of unknown significance (VUS) were further analysed with SpliceAI and CAPICE. Variants with a SpliceAI score (delta score) >0.42 were classified as pathogenic, and variants with a SpliceAI score ≤0.42 and a CAPICE score >0.56 were classified as LP. The remaining variants were classified as VUS.

MOLGENIS VIP can be downloaded from <https://github.com/molgenis/vip>.

1. **References**

1. Robinson PN, Ravanmehr V, Jacobsen JOB, Danis D, Zhang XA, Carmody LC, et al. Interpretable Clinical Genomics with a Likelihood Ratio Paradigm. Am J Hum Genet. 2020;107(3):403-17.

2. Smedley D, Jacobsen JO, Jager M, Kohler S, Holtgrewe M, Schubach M, et al. Next-generation diagnostics and disease-gene discovery with the Exomiser. Nat Protoc. 2015;10(12):2004-15.

3. Landrum MJ, Lee JM, Riley GR, Jang W, Rubinstein WS, Church DM, et al. ClinVar: public archive of relationships among sequence variation and human phenotype. Nucleic Acids Res. 2014;42(Database issue):D980-5.

4. Fokkema I, van der Velde KJ, Slofstra MK, Ruivenkamp CAL, Vogel MJ, Pfundt R, et al. Dutch genome diagnostic laboratories accelerated and improved variant interpretation and increased accuracy by sharing data. Hum Mutat. 2019;40(12):2230-8.

5. Karczewski KJ, Francioli LC, Tiao G, Cummings BB, Alfoldi J, Wang Q, et al. The mutational constraint spectrum quantified from variation in 141,456 humans. Nature. 2020;581(7809):434-43.

6. Li S, van der Velde KJ, de Ridder D, van Dijk ADJ, Soudis D, Zwerwer LR, et al. CAPICE: a computational method for Consequence-Agnostic Pathogenicity Interpretation of Clinical Exome variations. Genome Med. 2020;12(1):75.

7. Geoffroy V, Herenger Y, Kress A, Stoetzel C, Piton A, Dollfus H, et al. AnnotSV: an integrated tool for structural variations annotation. Bioinformatics. 2018;34(20):3572-4.

8. Amberger J, Bocchini CA, Scott AF, Hamosh A. McKusick's Online Mendelian Inheritance in Man (OMIM). Nucleic Acids Res. 2009;37(Database issue):D793-6.
