# Supplementary material for: Curation and expansion of the Human Phenotype Ontology for systemic autoinflammatory diseases improves phenotype-driven disease-matching
Source: Front Immunol. 2023 Sep 12;14:1215869. doi: 10.3389/fimmu.2023.1215869 (PMC10536149; doi:10.3389/fimmu.2023.1215869)
Supplement: Supplementary file 6 [file DataSheet_2.docx]

**Supplementary table**

File name: supplementary_table_1.xlsx

Supplementary table 1 **Number of patients collected from each expertise centre.** The first column lists the hospital where the patient information originates from. The second column shows the total number of patients with verified SAIDs that were part of the curation effort. The third column shows the number of patients for whom WES data is also available. The last column is the average number of documented HPO terms for each centre.

File name: supplementary_table_2.xlsx

Supplementary Table 2 **Diseases for which HPO terms were part of the study.** The first five columns contain the short names of the diseases, their long names, the disease ID in the OMIM database (1), the causal genes and the OMIM ID for the genes (1), respectively. The last column shows the number of patients in the study population with that specific diagnosis.

File name: supplementary_table_3.xlsx

Supplementary Table 3 **HPO terms for the 10 new reannotated diseases.** The first three columns contain the short names of the diseases, the gene symbols and the disease ID in the OMIM database (1). The last two columns show the number of HPO terms reannotated to each disease and the individual HPO terms, respectively.

File name: supplementary_table_4.xlsx

Supplementary Table 4 **Assigned HPO terms per included patient.** The first column contains the anonymized patient identifiers (numerical from 1 to 98). The second and the third column contain the short names of the diseases and the disease ID in the OMIM database (1). The last two columns show the number of HPO terms assigned to each patient and the individual HPO terms, respectively.

**References**

1. Amberger J, Bocchini CA, Scott AF, Hamosh A. McKusick's Online Mendelian Inheritance in Man (OMIM). Nucleic Acids Res. 2009;37(Database issue):D793-6.
