# Supplementary material for: Curation and expansion of the Human Phenotype Ontology for systemic autoinflammatory diseases improves phenotype-driven disease-matching
Source: Front Immunol. 2023 Sep 12;14:1215869. doi: 10.3389/fimmu.2023.1215869 (PMC10536149; doi:10.3389/fimmu.2023.1215869)
Supplement: Supplementary file 7 [file Image_1.pdf]

Supplementary figures

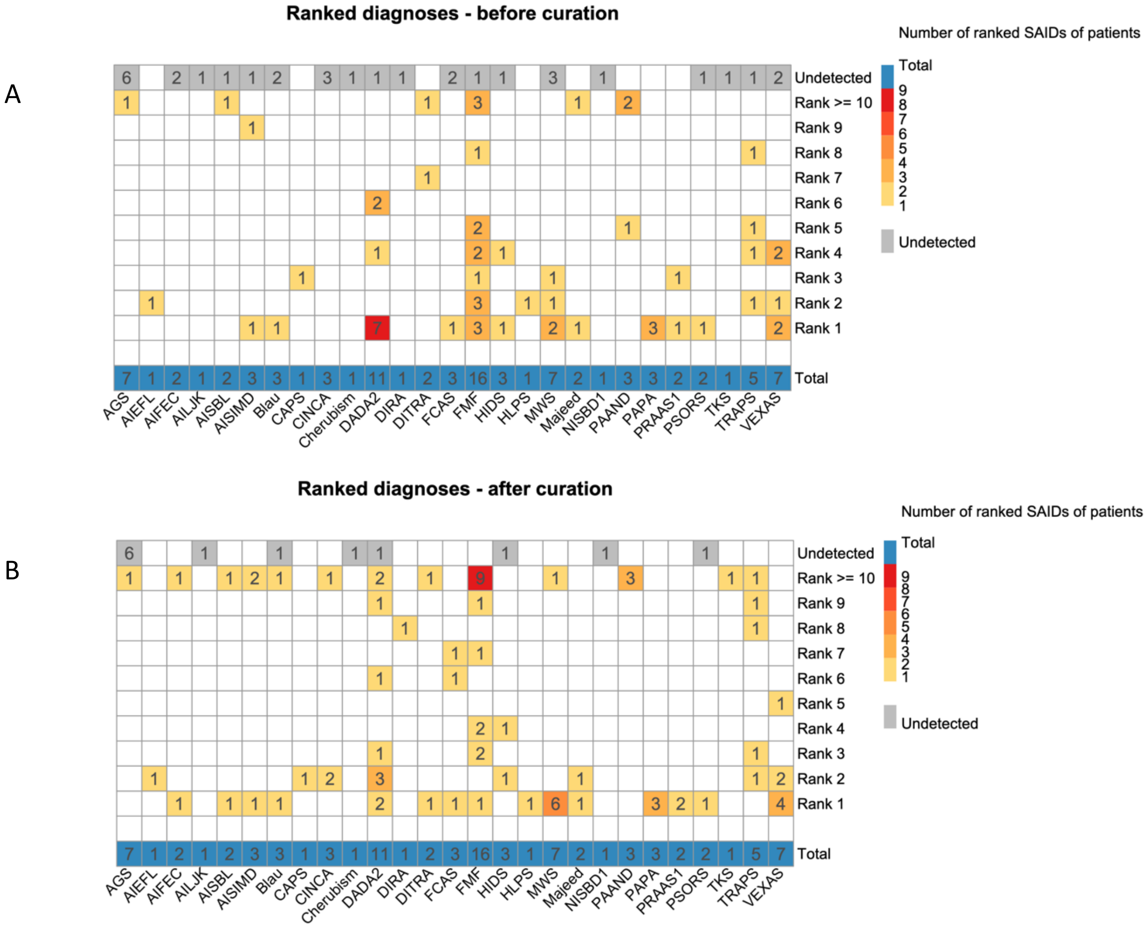

Supplementary figure 1 **Ranking of individual diseases before versus after curation of the HPO terms.** Both A and B are heatmaps showing the number of verified SAIDs assigned a rank from 1 to ≥10 (red to yellow). A) Ranked diagnoses before curation. B) Ranked diagnoses after curation. In both plots, the top row (grey) shows the number of SAIDs that were not detected with a LR > 0 and the bottom row (blue) shows the total number of verified cases.
